# I_2_BODIPY as a new photoswitchable spin label for light-induced pulsed EPR dipolar spectroscopy exploiting magnetophotoselection†

**DOI:** 10.1039/d4cp02297a

**Published:** 2024-11-06

**Authors:** Arnau Bertran, Susanna Ciuti, Daniele Panariti, Ciarán J. Rogers, Haiqing Wang, Jianzhang Zhao, Christiane R. Timmel, Marina Gobbo, Antonio Barbon, Marilena Di Valentin, Alice M. Bowen

**Affiliations:** a Centre for Advanced Electron Spin Resonance and Inorganic Chemistry Laboratory, University of Oxford South Parks Road Oxford OX1 3QR UK; b Department of Chemical Sciences, University of Padova Via Marzolo 1 Padova 35131 Italy antonio.barbon@unipd.it marilena.divalentin@unipd.it; c Department of Chemistry, Photon Science Institute and The National Research Facility for Electron Paramagnetic Resonance, University of Manchester Oxford Road Manchester M13 9PL UK alice.bowen@manchester.ac.uk; d State Key Laboratory of Fine Chemicals, Frontier Science Center for Smart Materials, School of Chemical Engineering, Dalian University of Technology Dalian 116024 P. R. China

## Abstract

Electron paramagnetic resonance (EPR) pulsed dipolar spectroscopy (PDS) using triplet states of organic molecules is a growing area of research due to the favourable properties that these transient states may afford over stable spin centers, such as switchability, increased signal intensity when the triplet is formed in a non-Boltzmann distribution and the triplet signal is used for detection, and high orientation selection, when the triplet signal is probed by microwave pulses. This arises due to the large spectral width at low fields, a result of the large zero field splitting, and limited bandwidth of microwave pulses used. Here we propose the triplet state of a substituted BODIPY moiety as a spin label in light induced PDS, coupled to a nitroxide, in a model peptide with a rigid structure. Orientation selection allows information on the relative position of the centres of the two labels to be obtained with respect to the nitroxide reference frame. Additionally, magnetophotoselection effects are employed to introduce optical selection and additional constraints for the determination of the relative orientation of the spin labels considering the reference frame of the triplet state.

## Abstract 2

We demonstrate that the triplet state of I_2_BODIPY can be employed as a spin label for EPR distance determination using magnetophotoselection for increased orientational selectivity.

## Graphical abstract

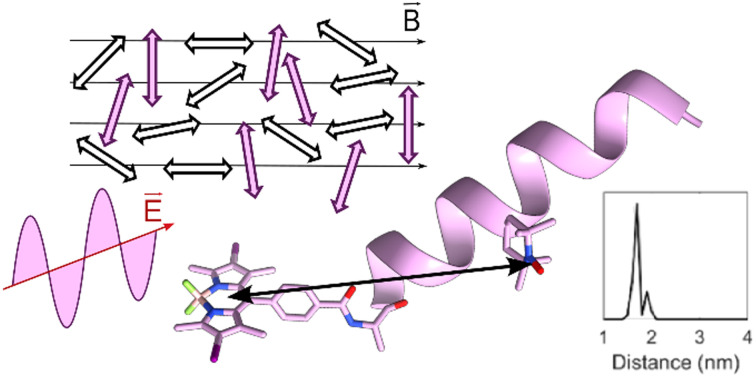

## Introduction

Electron paramagnetic resonance (EPR) pulsed dipolar spectroscopy (PDS) has become an important tool for studying the structure and dynamics of complex biomolecular assemblies.^1–5^ Using microwave pulses to measure the electron–electron dipolar interaction between two moieties with nonzero electronic spin, called spin labels, PDS techniques allow for the determination of the relative distance and, for rigid systems, orientation distributions, providing conformational information about the biomolecular assembly to which they are attached.^6–10^ The distance range of 1.5 to 8 nm,^11^ highly relevant in biological systems, can be accessed using typical PDS techniques, such as double electron–electron resonance (DEER), using conventional nitroxide spin labels introduced *via* site-directed mutagenesis or chemical modifications.^2,12,13^ Other spin labels, mainly trityl radicals^14,15^ and metal centres such as Gd(ii),^16^ Mn(ii)^17^ and Cu(ii),^18,19^ are emerging alternatives to nitroxide radicals, with controllable spectroscopic properties and improved stability.

In recent years a new type of spin label, the photoexcited triplet state of organic chromophores, has come into play, starting a paradigm shift in PDS.^20–26^ Instead of relying on several permanent open-shell paramagnetic centres such as the typically employed stable radicals, macromolecular systems with closed-shell labels can now be studied by EPR after electron spin-active triplet states are formed through laser excitation at an appropriate wavelength. In addition to being photoswitchable, these paramagnetic centres are formed in a spin-polarised state, leading to stronger EPR signals compared to those of Boltzmann-populated spin centres.^27,28^

Multiple light-induced PDS (LiPDS) techniques have been recently developed based on the photoexcited triplet state of 5(4′-carboxyphenyl)-10,15,20-triphenylporphyrin (TPP) incorporated into model peptide systems.^20–22,29–33^ Amongst LiPDS techniques, light-induced DEER (LiDEER)^20^ and laser-induced magnetic dipole spectroscopy (LaserIMD)^29^ are the most frequent choices to study the dipolar interaction between a photoexcited triplet and a stable radical, both in proteins^23,25,26,34^ and model peptides.^20,21,29–31,33^ Both techniques use a laser pulse to form the triplet state and microwave pulses to manipulate the spins, and their performance at different microwave frequencies (X-band and Q-band) has been previously compared.^30,31^ In the double-frequency experiment LiDEER, the photoexcited triplet formed by an initial laser flash is used as the detection spin while the dipolar modulation originates from the microwave-induced spin flip of the permanent radical. The technique has shown an accessible distance range similar to that of conventional PDS.^20^ Conversely, LaserIMD relies on optically switching the dipolar interaction by forming the triplet state while detecting the signal of the permanent radical.^29^ LaserIMD has the advantage of using a simpler and shorter single-frequency microwave pulse sequence with primary Hahn echo detection. However, the refocused echo version of this technique (ReLaserIMD) offers a more accurate determination of the zero time in the dipolar time traces and is preferable when studying short spin–spin distances.^22^

It has recently been shown that the anisotropy of the nitroxide spectrum at the Q-band can be exploited to obtain information on the relative orientation between the stable radical and the dipolar vector connecting it to the TPP triplet in a model peptide, by means of orientation-selective ReLaserIMD experiments.^33^ This was achieved both with multiple single-frequency 1D experiments, probing different parts of the nitroxide spectrum with the microwave pulses, and with a single frequency-correlated 2D experiment, where shaped pulses were used to cover the full spectral width of the nitroxide and obtain the entire orientational dataset in a single shot. The combination of these results with orientation-selective LiDEER experiments performed on different parts of the TPP triplet spectrum allowed pinpointing the relative orientation of the TPP moiety and rendered the distribution of molecular conformations in the frozen state, providing information on the dynamics of the peptide in solution. Both LaserIMD and LiDEER use spectroscopically orthogonal spin labels, the use of such combinations of spin labels has been of interest in biological systems as it makes it possible to know which spin label is attached at which site in the system.^35^

The search for new photoswitchable spin labels for LiPDS is an active area of research and a priority for the development of the field. Besides porphyrins, few other chromophores have been reported as labels for LiPDS, namely C_60_, eosin Y, rose bengal, ATTO Thio12 and erythrosin B.^23,26,36,37^ Good photoswitchable spin labels for LiPDS must have favourable optical, magnetic and photophysical properties, such as a high extinction coefficient in the visible range, high triplet quantum yield (*ϕ*_T_), favourable triplet lifetime, slow spin relaxation and good photostability.^36^ In addition, in order to be suitable for biological applications, the chromophores must be small and biocompatible.

**Fig. 1:**
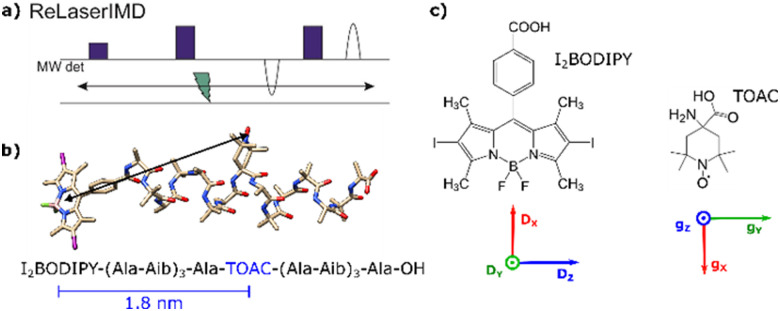
(a) ReLaserIMD pulse sequence. (b) Ground-state DFT-optimised geometry of 1 and corresponding amino acid sequence, indicating the inter-spin distance predicted by DFT. (c) Chemical structures of the two spin labels, with the principal axes of their ZFS and ***g***-tensors, respectively (red = *x*, green = *y*, blue = *z*). Amino acid key: Ala (l-alanine), Aib (α-aminoisobutyric acid) and TOAC (2,2,6,6-tetramethylpiperidine-1-oxyl-4-amino-4-carboxylic acid).

In this work, we introduce a new photoswitchable spin label for LiPDS, the photoexcited triplet state of a modified BODIPY chromophore. The I_2_BODIPY molecule, whose structure is shown in Fig. 1(c), is a derivative of BODIPY, obtained by halogenation, with a higher triplet yield than the corresponding BODIPY molecule.^38^ BODIPY is naturally a very fluorescent probe, unsensitive to photobleaching as compared, for example, to rose bengal.^38^ Most of its derivatives have a maximum absorption near 500 nm and high absorption coefficients. Introduction of substituents such as heavy atoms leads to high *ϕ*_T_^39^ and the quenching of fluorescence.^39,40^ Usable triplet lifetimes for LiPDS must be longer than the time window of dipolar interaction, but consequently can span several orders of magnitude, from few μs^39,41^ to hundreds or thousands of μs.^42^ For I_2_BODIPY, the triplet lifetime has been measured to be 122 μs at room temperature,^43^ while at 77 K it has been measured to be 1070 μs.^41^ Additionally, I_2_BODIPY has a high triplet quantum yield (*ϕ*_T_ = 88%), allowing for low-demanding photoexcitation.^39^ The large *ϕ*_T_ and reasonably long triplet lifetime make this compound suitable for LiPDS, where the high *ϕ*_T_ leads to greater experimental efficiency and where the triplet lifetime must be sufficiently longer than the recorded time trace. To study the application of I_2_BODIPY to LiPDS methods we use a rigid α-helical model peptide (1 in Fig. 1(b)), consisting of alternating l-alanine (Ala) and α-aminoisobutyric acid (Aib), bis-labelled with I_2_BODIPY and nitroxide in the form of 2,2,6,6-tetramethylpiperidine-1-oxyl-4-amino-4-carboxylic acid (TOAC).

We obtain orientation-selective dipolar datasets using the ReLaserIMD technique (Fig. 1(a)) at different field positions across the Q-band nitroxide spectrum with depolarised light. Orientation-dependent simulations assisted by density functional theory (DFT) calculations provide information on the orientation of the nitroxide radical relative to the dipolar vector connecting it to the chromophore. Unlike for porphyrins, the short phase memory time (*T*_m_) of the I_2_BODIPY triplet spins prevents the use of LiDEER to pinpoint the triplet orientation.^33^ Instead, we exploit magnetophotoselection effects on the triplet spectrum for the first time in LiPDS, to aid localisation of the orientation of the zero-field splitting (ZFS) tensor of the I_2_BODIPY chromophore relative to the ***g***-tensor frame of the nitroxide radical. Magnetophotoselection arises from the use of linearly polarised light to generate a triplet state of a chromophore in a magnetic field.^44^ The relative orientation of the transition dipolar moment (TDM) of the chromophore and the direction of polarisation of the light causes preferential formation of triplet states on chromophores with specific orientations with respect to the external magnetic field. Magnetophotoselection is usually observed in the line shape of the time-resolved electron paramagnetic resonance (trEPR) spectrum, which changes when light polarised perpendicular or parallel to the magnetic field direction is used. Here, we show computationally the effect that magnetophotoselection can have on LaserIMD traces, a result of orientation selection, similar to the consequences of incomplete excitation of an EPR spectrum by microwave pulses.^9,45^ By synergistically modelling the orientation-selection and magnetophotoselection effects in ReLaserIMD experiments, we determine the conformational distribution of the model compound in frozen solution, which is in good agreement with the DFT energy-minimised geometry.

## Results and discussion

*In vacuo* DFT optimisation predicts a I_2_BODIPY–nitroxide distance of 1.8 nm for bis-labelled model peptide system 1 (Fig. 1(b)), which is well within the measurement range of PDS. The chromophore was directly attached to the N-terminus of the peptide *via* amide bonding with the in-built carboxylic group of I_2_BODIPY (Fig. 1(c)), in order to minimise conformational flexibility. Details of the synthesis and purification of 1 are given in the ESI.†

### Orientation-selective ReLaserIMD

**Fig. 2:**
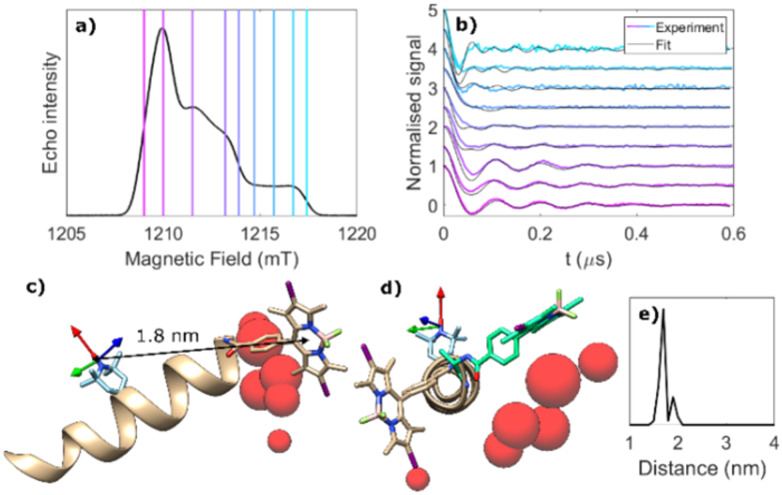
Orientationally selective ReLaserIMD on 1. (a) Electron spin-echo field-swept spectrum in the dark, showing the magnetic field values where ReLaserIMD traces were acquired. (b) Background-corrected and modulation depth-normalised ReLaserIMD traces (coloured lines) and orientation-dependent model-based fits (black lines). Modulation depths before normalisation were between 3 and 6%. (c) DFT-optimised structure of 1 showing the different positions of the I_2_BODIPY centre determined by the fitting procedure as red spheres, relative to the nitroxide ***g***-tensor frame (arrows: red = *g*_*x*_, green = *g*_*y*_, blue = *g*_*z*_). The diameter of the spheres is proportional to the number of times a single I_2_BODIPY position contributes to the fit shown in panel (b). (d) Projection view of panel (c), including a second local energy minimum conformation (+9 kJ mol^−1^) identified by DFT (green). (e) Distance distribution between the centres of the two spin-bearing moieties obtained from the orientation-dependent analysis.

Multiple ReLaserIMD (Fig. 1(a)) measurements were carried out using depolarised light at field values spanning the full width of the nitroxide EPR spectrum at the Q-band (Fig. 2(a)), yielding an orientation-resolved set of dipolar traces. The appearance of a faster dipolar frequency component with increasing external magnetic field clearly shows the presence of strong orientational effects due to orientation selection of the narrow microwave pulses, which are resonant with only a small fraction of the nitroxide orientations with respect to the magnetic field (Fig. 2(b), coloured lines). Parameters used for these measurements and the results are reported in line with the guidance for DEER results,^46^ and modified for light-induced PDS EPR methods^36,47^ in the ESI† (Section S3.3 and Table S4).

Orientation-dependent simulations, that consider ZFS in the spin Hamiltonian but only the secular parts of the dipolar interaction, were carried out using a previously published algorithm^8^ and were used to fit the experimental dataset in an iterative least-squares global fitting process.^48^ It has been shown that the non-secular parts of ZFS can lead to an additional decay in LaserIMD at low fields (X-band and lower), but this has little effect on LiDEER datasets.^49^ In the case of this analysis any additional decay in the LaserIMD traces due to non-secular effects was assumed to be small for the data measured at the Q-band, and treated as a background contribution which was removed using a non-homogeneous background correction. Additionally, unlike DEER and the related LiDEER technique, where the form of the background is dependent only on the inter-molecular interactions caused by the relative distributions of molecules in the sample,^50^ in LaserIMD methods the time-variant increase of spin active species upon laser excitation means that the relaxation time of the spin centre used for detection changes upon the generation of the triplet state and thus there is likely an additional relaxation-induced component to the LaserIMD background function that is absent in the background of PDS experiments where the number of spin active moieties is constant during the period of dipolar evolutions, such as DEER. A set of geometric models of the relative positions of the nitroxide and I_2_BODIPY using the DFT-optimised minimum energy conformation of 1 as the centre of the distribution, were generated. Simulations of LaserIMD traces included the electron spin density distributions of the I_2_BODIPY triplet and the nitroxide radical calculated by DFT (Fig. S9, ESI†). The geometric space covered by this model was described by a spherical coordinate system (*ϕ*, *θ*, *r*), where *ϕ*, *θ* were varied over 30°, and *r* by 0.2 nm, and a set of Euler angles (*α*, *β*, *γ*) to describe relative orientations of the nitroxide molecular and ***g***-tensor frame and the I_2_BODIPY molecular and ZFS tensor frame, varying each by 20°. After initially fitting the traces simulated to the experimental data, a finer grid around the initial best fit was produced and added to the model. Complete details of the model used, the simulation and the fitting procedures are given in the ESI† (Section S2.2).

The fitted traces from this model provide a good description of the experimental dipolar data, capturing the orientational effects on the dipolar frequencies (Fig. 2(b), black lines). The corresponding distance distribution between the centres of the two chromophores in the models used to generate the fitted traces is sharp, and in perfect agreement with the nitroxide-to-chromophore–centre distance in the lowest-energy calculated DFT structure of 1.8 nm (Fig. 2(e)). The molecular conformational information obtained from the model-based orientation-selective fit is shown in Fig. 2(c) as a distribution of the I_2_BODIPY chromophore position with respect to the fixed ***g***-tensor frame of the nitroxide radical.^51^ The red spheres represent the positions of the centre of the I_2_BODIPY chromophore contributing to the best fit shown in Fig. 2(b), with the diameter of each sphere being proportional to the weight of the contribution of that particular chromophore position to the overall fit. The resulting conformational distribution is close to the DFT minimum-energy structure, but it suggests some degree of rotational flexibility around the N-terminus of the peptide. Indeed, higher local energy minimum conformations were identified by DFT through rotation of the C–C bond of the first Ala residue (Fig. 2(d), green structure). DFT calculations were performed *in vacuo* and therefore do not capture any solvent effects that may influence the orientation of the I_2_BODIPY chromophore relative to the peptide helix. The helix is formed of both Ala and Aib residues; Aib has been shown to have a high hydrophobicity index compared to Ala,^45^ and consequently the I_2_BODIPY chromophore may orientate to partially protect the Aib residue at the N-terminus.

A second, model-free analysis of the dipolar dataset was performed allowing unrestricted conformational flexibility of the position of the chromophore relative to the nitroxide ***g***-tensor frame (Fig. S10, ESI†) using a spherical grid over one-quarter of a sphere with *C*_1_ symmetry and a knot every 10°, and *r* varying from 1.5 nm to 2.1 nm in 0.1 nm increments. Due to the higher computational cost of this method, the electron spin density of the I_2_BODIPY triplet was considered to be concentrated at a single point. The model-free approach also provided a very good description of the experimental data, with results showing a spread of geometric distribution, compared to the DFT model-based calculation (Fig. S10, ESI†). In both calculations the main source of distributions originates from the rotational flexibility at the N-terminus.

### Magnetophotoselection ReLaserIMD

**Fig. 3:**
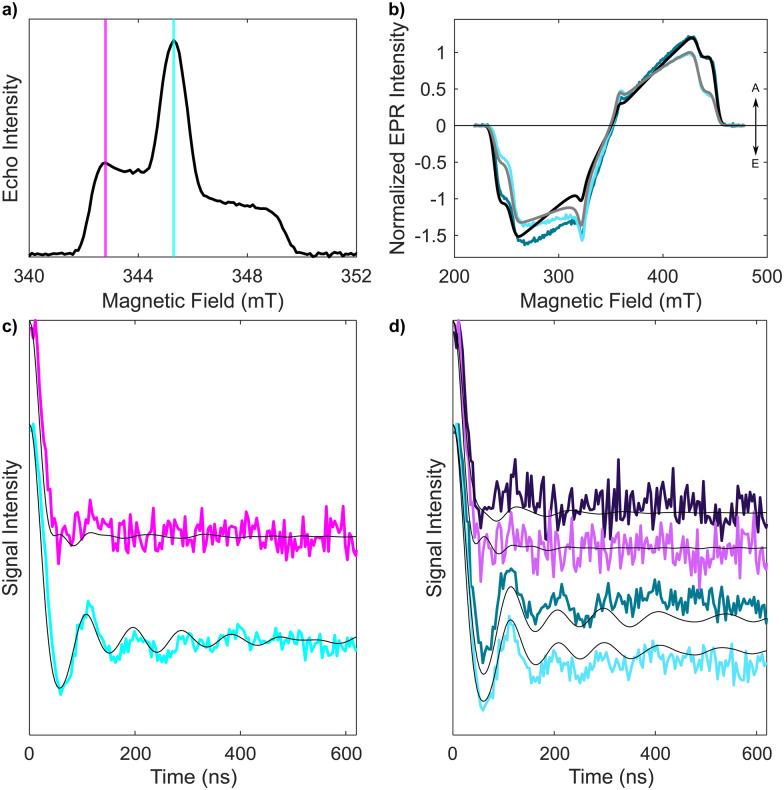
X-band EPR experiments on 1 with linearly polarised light. (a) Electron spin-echo field-swept spectrum in the absence of photoexcitation, showing the magnetic field values where ReLaserIMD traces were acquired. (b) Experimental trEPR spectra of I_2_BODIPY, at the maximum of the triplet signal recorded in the time domain, obtained with light polarised parallel (darker blue) and perpendicular (lighter blue) with respect to the magnetic field, and the corresponding simulations (black and grey, respectively). Relative intensities of the spectra with light polarized parallel or perpendicular to the magnetic field are maintained for both experimental and simulated traces, while the maximum of the one obtained for the perpendicular configuration is set to 1. Simulation parameters are reported in Table S4 (ESI†). (c) Experimental magnetophotoselection-free ReLaserIMD form factors (colored lines), generated from averaging the traces (measured at the field positions indicated in panel (a)) with perpendicular and parallel linearly polarized light according to eqn (4), with normalized modulation depths. Corresponding simulations (black lines) obtained using the X-band EPR experimental parameters and geometrical parameters of the best fitting model obtained from the Q-band data. (d) Experimental form factors of the ReLaserIMD traces obtained at 345.3 mT (blue traces, *m*_I_ = 0) and 342.8 mT (purple traces, *m*_I_ = +1) with light polarised parallel (darker colors) and perpendicular (lighter colors) to the magnetic field and corresponding simulations (black). The modulation depths for the *m*_I_ = 0 and *m*_I_ = +1 datasets were corrected using the normalization factors applied to the data in panel (c).

Initial tests to study the magnetophotoselection of the I_2_BODIPY chromophore bound to the peptide using trEPR at X-band (9.7 GHz) and Q-band (34 GHz) frequencies showed that the magnetophotoselection effects were significantly stronger in the X-band datasets. This might be due in part to additional light scattering at the Q-band due to the smaller tubes used, 3 mm outer diameter (O.D.) at the Q-band and 4 mm O.D. at the X-band, or an effect of the differing geometries and materials of the resonators on the scattering of light within them. EPR data recorded at the X-band with and without polarised light are depicted in Fig. 3. Simulations of the trEPR spectra with light linearly polarised perpendicular and parallel to the magnetic field, shown in Fig. 3(b), were recorded using TESEO,^52^ which employs expressions for the excitation probability *p*(*α,β,ω,φ*) (eqn (1) and (2)), derived by Toffoletti *et al.*:^44^1
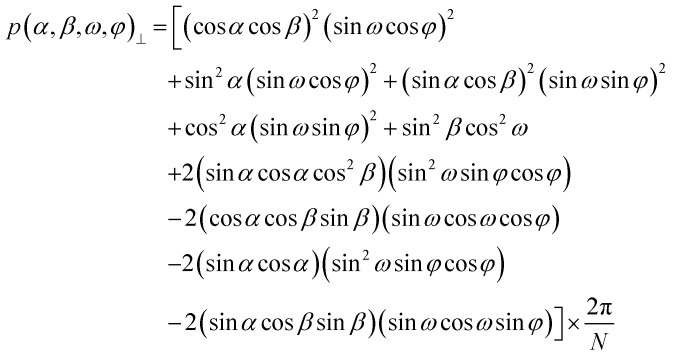
2
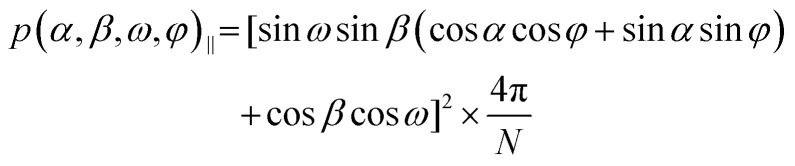
In these expressions, the angles *ω* and *φ* describe the tilt of the TDM relative to the principal axes of the ZFS tensor frame. *α* and *β*, which describe the orientation of the magnetic field relative to the molecule, have been varied over the entire geometrical parameter space to provide a complete orientational averaging for the simulations. Simulations of the trEPR (Fig. 3(b)) data yielded good descriptions of the experimental spectra using *ω* = 22° and *φ* = 0°, which is in good agreement with the parameters defined from the related I-BODIPY molecule with a single iodo substitution.^44^

The dipolar traces obtained with polarized light after background correction, reported in Fig. 3(d), show differences in both modulation depth and dipolar oscillations (see also Fig. S13, ESI†). The parameters used for these measurements and the corresponding results are reported in line with guidance for DEER results,^46^ and modified for light-induced PDS EPR methods,^36,47^ in the ESI† (Section S3.3 and Table S5). Several background models were trialled for the X-band ReLaserIMD traces. Results indicated that good matches with data recorded at Q-band, in terms of the observed frequencies at the field positions shown in Fig. 3(a), could be obtained from background correction using a three-dimensional exponential (to remove the intermolecular dipolar interaction background components) followed by a fourth-order polynomial background correction to account for relaxation and ZFS non-secular contributions (Fig. S14, ESI†).

When taking into account the polarization of the light used for photoexcitation of chromophores, it is known, both geometrically^53^ and from experimental trEPR data,^54^ that a spectrum free from magnetophotoselection (*I*_avg_) can be obtained by combining the datasets recorded with light polarised perpendicular (*I*_⊥_) and parallel (*I*_‖_) to the magnetic field according to:

3 *I*_avg_ = (2*I*_⊥_ + *I*_‖_)/3

In LaserIMD traces recorded with polarised light, magnetophotoselection affects the modulation depth rather than the echo intensity, as this is dependent on the permanent spin centre (the nitroxide in this case) and the field position used and not on the light-activated chromophore. The normalised form factors measured with perpendicular and parallel light polarisation with respect to the magnetic field (*F*_⊥_ and *F*_‖_, respectively) can be combined to obtain the form factor without magnetophotoselection (*F*_avg_), with a modulation depth that corresponds to that of the weighted sum of the ones obtained in the two polarisation conditions using the following equation:

4 *F*_avg_ = (2*F*_⊥_ + *F*_‖_) − 2

After background correction of the experimental data the resulting form factors were combined according to eqn (4) to generate traces free of magnetophotoselection effects (see Fig. 3(c)). In the absence of magnetophotoselection effects the modulation depth of the traces would be expected to be independent of field position, as all orientations of the I_2_BODIPY molecule will be excited. However, experimentally it was observed that the average magnetophotoselection-free traces measured on the two different transitions (*m*_I_ = 0 and *m*_I_ = +1) had different modulation depths. It is noted that the modulation depth can be affected by the photoexcitation conditions. These include the quality of the sample glass (as can be seen by different modulation depths in data recorded on different days), which affects the penetration depth of light in the sample, and the achievable laser power at the sample, as some power is lost due to reflection/scattering at the cryostat optical window which can be variable over time if ice builds up. In addition, laser power in prolonged measurements may be affected by instabilities. To take into account the different photoexcitation conditions, the modulation depth of the polarization averaged form factors, were corrected to the same value. The same correction factors were afterwards applied to the individual form factors for the traces measured with polarised light. The averaged magnetophotoselection-free experimental traces (Fig. 3(c) coloured lines) were compared to simulated traces (black lines) calculated using the geometric parameters from the best fitting model derived from the Q-band data (presented in Fig. 2) and the experimental parameters used to record the X-band traces. For the modulation depth-corrected polarised traces (Fig. 3(d)), it was observed that the trace recorded with perpendicularly polarised light had a larger modulation depth for both the datasets measured on the *m*_I_ = 0 and +1 transitions (Fig. S13, ESI†). Additionally, the difference in modulation depth between the perpendicular and parallel polarization was larger for datasets recorded on the *m*_I_ = 0 transition than on the *m*_I_ = +1 transition. This gave an experimental ordering of modulation depths (*Δ*) of Δ(*m*_I_ = 0,⊥) > Δ(*m*_I_ = +1,⊥) > Δ(*m*_I_ = +1,‖) > Δ(*m*_I_ = 0,‖).

A previously published algorithm for calculating orientationally selective PDS traces^8^ was modified to include the excitation probability functions for light polarised parallel and perpendicular to the magnetic field (eqn (1) and (2)), using the *EasySpin* ordering function to interface with the existing orientationally selective PDS simulation program,^55^ allowing for the simulation of LiPDS traces with magnetophotoselection. This program is included in the data repository (see the Acknowledgements section for details). From the model-based simulation it was found that *ca.* 58% of the parameter space covered by the model fitted to the Q-band data could be described by five conformations, represented by the five largest spheres in Fig. 2(c). For each conformation the centre of the I_2_BODIPY chromophore is described by a fixed set of spherical polar coordinates (*ϕ*, *θ*, *r*). Simulations of dipolar traces including magnetophotoselection were carried out for a complete parameter space of Euler angles (*α*, *β*, *γ*) describing the orientation of the I_2_BODIPY molecular and ZFS tensor frame relative to the nitroxide (***g***-tensor) frame, further details are provided in the ESI† (Section S2.3). The results of these calculations showed variations in both the modulation depth and oscillation frequencies with different Euler angles (*α*, *β*, *γ*). As the oscillation frequencies are also sensitive to the spherical polar coordinates (*ϕ*, *θ*, *r*) and the largest variation in the simulated traces with (*α*, *β*, *γ*) was observed in the modulation depth, the latter was chosen as the primary analysis measure.

Sets of simulated traces calculated with the same Euler angles (*α*, *β*, *γ*) which obeyed the modulation depth ordering Δ(*m*_I_ = 0,⊥) > Δ(*m*_I_ = +1,⊥) > Δ(*m*_I_ = +1,‖) > Δ(*m*_I_ = 0,‖) were selected and the traces corresponding to each set of spherical polar coordinates (*ϕ*, *θ*, *r*) were summed using a weighting factor based on their contribution to the model presented in Fig. 2, generating the black simulated traces shown in Fig. 3(d). As a result, it was possible to restrict the parameter space in which the principal *z*-axis of the ZFS tensor of the I_2_BODIPY can lie relative to the nitroxide ***g***-tensor frame and the dipolar vector (Fig. S15, ESI†). The results show the predicted possible *z*-axis orientations of the ZFS tensor, parallel to the long axis of I_2_BODIPY, which are in very good agreement with respect to the molecular structure of 1 predicted by DFT optimisation.

## Conclusions

We have demonstrated that I_2_BODIPY is a suitable chromophore for application as a photoactivated spin label in LaserIMD experiments coupled with a nitroxide spin centre and that strong orientation selection can be observed in the traces recorded across the nitroxide spectrum at the Q-band, providing information about the relative orientation of the dipolar vector with respect to the nitroxide ***g***-tensor and the distance between the spin centres.

Furthermore, additional information can be gained about the relative orientation of the nitroxide ***g***-tensor and ZFS tensor of the chromophore using differences in modulation depth in traces recorded with either perpendicular or parallel polarisation of excitation light. This is an important step towards demonstrating selectivity with light in PDS EPR. This work also demonstrates the requirement for a depolariser to be included in the beam path for experiments recorded at the X-band if magnetophotoselection effects are not desired.

## Author contributions

Conceptualisation, A. Barbon, M. D. V. and A. M. B.; methodology, A. Bertran, A. Barbon, M. D. V. and A. M. B.; software, A. Barbon and A. M. B.; formal analysis, A. Bertran, S. C., D. P., C. J. R., A. Barbon and A. M. B.; investigation, A. Bertran, S. C., D. P., M. G., H. W., J. Z. and A. M. B.; resources, M. G., C. R. T., A. Barbon, M. D. V. and A. M. B.; writing – original draft, A. Bertran and A. M. B.; writing – review and editing, A. Bertran, M. G., S. C., C. R. T., A. Barbon, M. D. V. and A. M. B.; visualisation, A. Bertran, S. C. and A. M. B.; supervision, C. R. T., A. Barbon, M. D. V. and A. M. B.; funding acquisition, M. G., C. R. T., A. Barbon, M. D. V. and A. M. B. All authors have read and agreed to the published version of the manuscript.

## Data availability

For the purpose of open access, the author has applied a Creative Commons Attribution (CC BY) license (where permitted by UKRI, ‘Open Government License’ or ‘Creative Commons Attribution No-derivatives (CC BY-ND) license may be stated instead) to any author accepted manuscript version arising. Data supporting this article have been included as part of the ESI.† Experimental data and analysis programs have been archived in the data repository with following DOIs (README: DOI: 10.48420/25343338; DFT calculations: DOI: 10.48420/25343398; spectroscopic characterisation: DOI: 10.48420/25376464; depolarised-light ReLaserIMD: DOI: 10.48420/25343506; polarised-light ReLaserIMD: DOI: 10.48420/25343440; magnetophotoselection simulations with polarised light: DOI: 10.48420/25914619).

## Conflicts of interest

There are no conflicts to declare.

## Supplementary Material

CP-026-D4CP02297A-s001

## References

[cit1] Schiemann O., Prisner T. F. (2007). Q. Rev. Biophys..

[cit2] Jeschke G. (2012). Annu. Rev. Phys. Chem..

[cit3] Borbat P. P. and Freed J. H., in Structural Information from Spin-Labels and Indtrinsic Paramagnetic Centres in the Biosciences, ed. C. R. Timmel and J. R. Harmer, Springer-Verlag, Berlin and Heidelberg, Berlin, 2013, pp. 1–82

[cit4] Jeschke G. (2018). Emerging Top. Life Sci..

[cit5] Goldfarb D. (2019). J. Magn. Reson..

[cit6] Denysenkov V. P., Prisner T. F., Stubbe J., Bennati M. (2006). Proc. Natl. Acad. Sci. U. S. A..

[cit7] Bode B. E., Plackmeyer J., Prisner T. F., Schiemann O. (2008). J. Phys. Chem. A.

[cit8] Lovett J. E., Bowen A. M., Timmel C. R., Jones M. W., Dilworth J. R., Caprotti D., Bell S. G., Wong L. L., Harmer J. (2009). Phys. Chem. Chem. Phys..

[cit9] Bowen A. M. , Tait C. E., Timmel C. R. and Harmer J. R., in Structural Information from Spin-Labels and Intrinsic Paramagnetic Centres in the Biosciences, ed. C. R. Timmel and J. R. Harmer, Springer, Berlin, Heidelberg, 1st edn, 2013, pp. 283–327

[cit10] Gamble Jarvi A., Ranguelova K., Ghosh S., Weber R. T., Saxena S. (2018). J. Phys. Chem. B.

[cit11] Banham J. E., Baker C. M., Ceola S., Day I. J., Grant G. H., Groenen E. J. J., Rodgers C. T., Jeschke G., Timmel C. R. (2008). J. Magn. Reson..

[cit12] Jeschke G. (2002). Chem. Phys. Chem..

[cit13] Jeschke G., Polyhach Y. (2007). Phys. Chem. Chem. Phys..

[cit14] Joseph B., Tormyshev V. M., Rogozhnikova O. Y., Akhmetzyanov D., Bagryanskaya E. G., Prisner T. F. (2016). Angew. Chem., Int. Ed..

[cit15] Jassoy J. J., Berndhäuser A., Duthie F., Kühn S. P., Hagelueken G., Schiemann O. (2017). Angew. Chem., Int. Ed..

[cit16] Goldfarb D. (2014). Phys. Chem. Chem. Phys..

[cit17] Banerjee D., Yagi H., Huber T., Otting G., Goldfarb D. (2012). J. Phys. Chem. Lett..

[cit18] Cunningham T. F., Putterman M. R., Desai A., Horne W. S., Saxena S. (2015). Angew. Chem., Int. Ed..

[cit19] Wort J. L., Ackermann K., Giannoulis A., Stewart A. J., Norman D. G., Bode B. E. (2019). Angew. Chem., Int. Ed..

[cit20] Di Valentin M., Albertini M., Zurlo E., Gobbo M., Carbonera D. (2014). J. Am. Chem. Soc..

[cit21] Di Valentin M., Albertini M., Dal Farra M. G., Zurlo E., Orian L., Polimeno A., Gobbo M., Carbonera D. (2016). Chem. - Eur. J..

[cit22] Dal Farra M. G., Richert S., Martin C., Larminie C., Gobbo M., Bergantino E., Timmel C. R., Bowen A. M., Di Valentin M. (2019). Chem. Phys. Chem..

[cit23] Krumkacheva O. A., Timofeev I. O., Politanskaya L. V., Polienko Y. F., Tretyakov E. V., Rogozhnikova O. Y., Trukhin D. V., Tormyshev V. M., Chubarov A. S., Bagryanskaya E. G., Fedin M. V. (2019). Angew. Chem..

[cit24] Serrer K., Matt C., Sokolov M., Kacprzak S., Schleicher E., Weber S. (2019). Mol. Phys..

[cit25] Sannikova N. E., Timofeev I. O., Chubarov A. S., Lebedeva N. S., Semeikin A. S., Kirilyuk I. A., Tsentalovich Y. P., Fedin M. V., Bagryanskaya E. G., Krumkacheva O. A. (2020). J. Photochem. Photobiol., B.

[cit26] Williams L., Tischlik S., Scherer A., Fischer J. W. A., Drescher M. (2020). Chem. Commun..

[cit27] Kay C. W. M., Di Valentin M., Möbius K. (1995). Sol. Energy Mater. Sol. Cells.

[cit28] Lubitz W., Lendzian F., Bittl R. (2002). Acc. Chem. Res..

[cit29] Hintze C., Bücker D., Domingo Köhler S., Jeschke G., Drescher M. (2016). J. Phys. Chem. Lett..

[cit30] Bieber A., Bücker D., Drescher M. (2018). J. Magn. Reson..

[cit31] Dal Farra M. G., Ciuti S., Gobbo M., Carbonera D., Di Valentin M. (2019). Mol. Phys..

[cit32] Bertran A., Henbest K. B., De Zotti M., Gobbo M., Timmel C. R., Di Valentin M., Bowen A. M. (2021). J. Phys. Chem. Lett..

[cit33] Bowen A. M., Bertran A., Henbest K. B., Gobbo M., Timmel C. R., Di Valentin M. (2021). J. Phys. Chem. Lett..

[cit34] Di Valentin M., Dal Farra M. G., Galazzo L., Albertini M., Schulte T., Hofmann E., Carbonera D. (2016). Biochim. Biophys. Acta, Bioenerg..

[cit35] Kaminker I., Yagi H., Huber T., Feintuch A., Otting G., Goldfarb D. (2012). Phys. Chem. Chem. Phys..

[cit36] Bertran A., Barbon A., Bowen A. M., Di Valentin M. (2022). Methods Enzymol..

[cit37] Bertran A., Morbiato L., Aquilia S., Gabbatore L., De Zotti M., Timmel C. R., Di Valentin M., Bowen A. M. (2022). Molecules.

[cit38] Loudet A., Burgess K. (2007). Chem. Rev..

[cit39] Zhao J., Xu K., Yang W., Wang Z., Zhong F. (2015). Chem. Soc. Rev..

[cit40] Imran M., Zhang X., Wang Z., Chen X., Zhao J., Barbon A., Voronkova V. K. (2021). Phys. Chem. Chem. Phys..

[cit41] Wang Z., Toffoletti A., Hou Y., Zhao J., Barbon A., Dick B. (2021). Chem. Sci..

[cit42] Wang Z., Sukhanov A. A., Toffoletti A., Sadiq F., Zhao J., Barbon A., Voronkova V. K., Dick B. (2019). J. Phys. Chem. C.

[cit43] Mahmood Z., Toffoletti A., Zhao J., Barbon A. (2017). J. Lumin..

[cit44] Toffoletti A., Wang Z., Zhao J., Tommasini M., Barbon A. (2018). Phys. Chem. Chem. Phys..

[cit45] Gadais C., Devillers E., Gasparik V., Chelain E., Pytkowicz J., Brigaud T. (2018). ChemBioChem.

[cit46] Schiemann O., Heubach C. A., Abdullin D., Ackermann K., Azarkh M., Bagryanskaya E. G., Drescher M., Endeward B., Freed J. H., Galazzo L., Goldfarb D., Hett T., Esteban Hofer L., Fábregas Ibáñez L., Hustedt E. J., Kucher S., Kuprov I., Lovett J. E., Meyer A., Ruthstein S., Saxena S., Stoll S., Timmel C. R., Di Valentin M., Mchaourab H. S., Prisner T. F., Bode B. E., Bordignon E., Bennati M., Jeschke G. (2021). J. Am. Chem. Soc..

[cit47] Bertran A., De Zotti M., Timmel C. R., Di Valentin M., Bowen A. M. (2024). Phys. Chem. Chem. Phys..

[cit48] Marko A., Prisner T. F. (2013). Phys. Chem. Chem. Phys..

[cit49] Scherer A., Yildirim B., Drescher M. (2023). Magn. Reson..

[cit50] Fábregas Ibáñez L., Jeschke G. (2020). Phys. Chem. Chem. Phys..

[cit51] Savitsky A., Dubinskii A. A., Plato M., Grishin Y. A., Zimmermann H., Möbius K. (2008). J. Phys. Chem. B.

[cit52] Strzelczyk R., Ciuti S., Carella A., Bortolus M., Franco L., Zoleo A., Ruzzi M., Toffoletti A., Di Valentin M., Carbonera D., Barbon A. (2023). Appl. Magn. Reson..

[cit53] Lakowicz J. R. , Principles of Fluorescence Spectroscopy, Springer, US, Boston, MA, 2006

[cit54] Barbon A., Dal Farra M. G., Ciuti S., Albertini M., Bolzonello L., Orian L., Di Valentin M. (2020). J. Chem. Phys..

[cit55] Tait C. E., Krzyaniak M. D., Stoll S. (2023). J. Magn. Reson..

