# Supplementary material for: I2BODIPY as a new photoswitchable spin label for light-induced pulsed EPR dipolar spectroscopy exploiting magnetophotoselection
Source: Phys Chem Chem Phys. 2024 Nov 6;26(45):28398–405. doi: 10.1039/d4cp02297a (PMC11563202; doi:10.1039/d4cp02297a)
Supplement: CP-026-D4CP02297A-s001 [file CP-026-D4CP02297A-s001.pdf]

This journal is © the Owner Societies 2024

Supplementary Materials for

I_2BODIPY as a new photoswitchable spin label for light-induced pulsed EPR dipolar spectroscopy exploiting magnetophotoselection

Arnau Bertran,[a†] Susanna Ciuti,[b,c†] Daniele Panariti,[b] Ciarán J. Rogers[c] Haiqing Wang,[d] Jianzhang Zhao,[d] Christiane R. Timmel,[a] Marina Gobbo,[b] Antonio Barbon,[b] Marilena Di Valentin[b*] and Alice M. Bowen[c*]*

[a] *Center for Advanced Electron Spin Resonance and Inorganic Chemistry Laboratory, Department of Chemistry, University of Oxford, South Parks Road, Oxford OX1 3QR, United Kingdom.*

[b] *Department of Chemical Sciences, University of Padova, Via Marzolo 1, 35131 Padova, Italy.*

[c] *Department of Chemistry, Photon Science Institute and The National Research Facility for Electron Paramagnetic Resonance, University of Manchester, Oxford Road, Manchester M13 9PL, United Kingdom.*

[d] *State Key Laboratory of Fine Chemicals, Frontier Science Center for Smart Materials, School of Chemical Engineering, Dalian University of Technology, Dalian 116024, P. R. China.*

* antonio.barbon@unipd.it, marilena.divalentin@unipd.it, alice.bowen@manchester.ac.uk.

† These authors contibuted equally to this work.

CONTENTS

S1. Experimental methods

S1.1. Synthetic methods

S1.2. Sample preparation

S1.3. EPR spectroscopy

S2. Computational methods

S2.1. DFT calculations

S2.2. Orientation-dependent ReLaserIMD simulations with depolarised light

S2.3. Orientation-dependent ReLaserIMD simulations with linearly polarised light

S3. Results

S3.1. Characterisation

S3.2. DFT results

S3.3. Orientation-selective ReLaserIMD

S3.4. Magnetophotoselection ReLaserIMD

REFERENCES

S1. EXPERIMENTAL METHODS

S1.1. Synthetic methods

Synthesis of I_2BODIPY N-succinimidyl ester (I_2BODIPY-ONSu)

I_2BODIPY-O*N*Su was synthesized as previously reported.[1] The mixture of I_2BODIPY-carboxy (50.0 mg, 0.081 mmol), EDC·HCl (34.5 mg, 0.18 mmol) and *N*-hydroxysuccinimide (20.7 mg, 0.18 mmol) was dissolved in dichloromethane (DCM. 20 mL). The mixture was stirred for 8 h at room temperature under N_2. The reaction mixture was washed with water (2 × 50 mL). The organic layer was dried over anhydrous Na_2SO_4. The solvent was evaporated under reduced pressure. The crude product was purified by column chromatography (silica gel, CH_2Cl_2). The product was obtained as a dark red solid (28 mg, yield 48%). ^1H NMR (400 MHz, $CDCl_3$) δ 8.32–8.30 (d, 2H, J = 8.0 Hz), 7.49–7.47 (d, 2H, J = 8.0 Hz), 2.96 (s, 4H), 2.66 (s, 6H), 1.39 (s, 6H). HRMS (MALDI): m/z calcd for $[C_{24}H_{20}BF_2I_2N_3O_4]^+$: 716.9604; found: 716.9586.

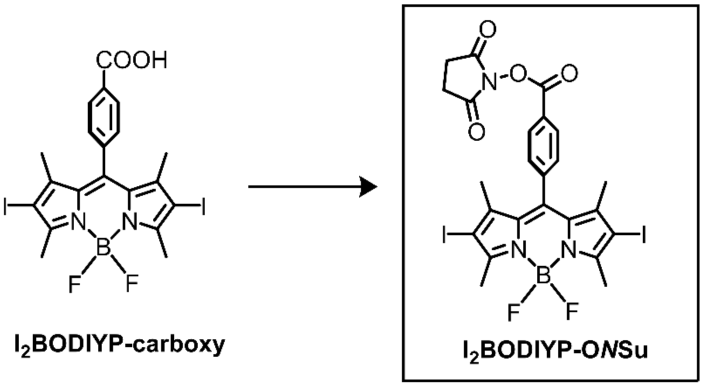

Scheme S1. Synthesis of I_2BODIPY-O*N*Su.

*Synthesis of I_2BODIPY-(Ala-Aib)$_3$-Ala-TOAC-(Ala-Aib)$_3$-Ala-OH (**1**)*

All chemicals were commercial products of the best grade available and, unless otherwise indicated they were used directly without further purification. 9-Fluorenylmethoxycarbonyl(Fmoc)-amino acids and all other chemicals for the solid phase synthesis were supplied by Sigma-Aldrich. Fmoc-4-amino-1-oxyl-2,2,6,6,-tetramethylpiperidine-4-carboxylic acid (TOAC) and H-Ala-2-Chlorotrityl resin were purchased from Iris Biotech (Germany). Analytical HPLC separations were carried out on a Dionex Summit Dual-Gradient HPLC, equipped with a four-channel UV-vis detector, using a Phenomenex Jupiter C4 column (250 x 4.6 mm, 5 μm, flow rate at 1.5 mL/min). The mobile phase A (aqueous 0.1% trifluoroacetic acid (TFA)) and B (90% aqueous acetonitrile containing 0.1% TFA) were used for preparing binary gradients. Mass spectral analysis was carried out on a Xevo G2-S QTof (Waters) operating with ESI technique in positive mode.

The peptide sequence was assembled on an automated Advanced Chemtech 348Ω Peptide Synthesizer, starting from H-Ala-2-Chlorotrityl resin (substitution 0.77 mmol/g resin), as previously reported [2]. 0.20 μmol of H-peptide-resin was swelled in DMF and a solution of I_2BODIPY-O*N*Su (0.40 μmol in DMF-CH_2Cl_2, 1:1 v/v) was added manually. The reaction mixture was shaken overnight, after that the resin was repeatedly washed with DMF and CH_2Cl_2 until the filtrate was colorless. The conjugate was cleaved from the resin upon 3-4 treatments with 30% 1,1,1,3,3,3-hexafluoropropan-2-ol in CH_2Cl_2 for 30 min. The filtrates

were combined and evaporated to dryness providing the conjugate, with an intact nitroxylic radical, that was characterized as follow.

HPLC (Jupiter C4; elution condition: isocratic 50% B 3 min; linear gradient 50-100% B in 30 min.) t_R 20.6 min (Fig. S3).

ESI-MS: calcd for $C_{78}H_{117}N_{18}O_{18}BF_2I_2$ $[M+H]^+$ 1896.68 m/z; found: 1896.69 m/z (Fig. S4).

S1.2. Sample preparation

Samples for Q-band EPR experiments were prepared to 50 µM in d_4-methanol:d_6-ethanol 3:2 (v:v) in quartz tubes (25 µL in 2.8 mm outer diameter 2.2 mm inner diameter tubes, sample height ~ 6 mm). The sample of I_2BODIPY for X-Band measurement was prepared to 15 µM in methanol:ethanol 2:3 (v:v). The sample of **1** for X-band experiments was prepared to 60 µM in d_4-methanol:d-glycerol 9:1 (100 µL in 4 mm outer diameter tubes). Samples were degassed by three freeze-pump-thaw cycles, flame-sealed and frozen in liquid nitrogen prior to insertion into the spectrometer. The degassing procedure was used to remove oxygen to maintain the stability of chromophore under the conditions of illumination.

S1.3. EPR spectroscopy

Experiments with depolarised light

Pulsed experiments with depolarised light were conducted in an Elexsys E580 spectrometer (Bruker) fitted with a spinjet Arbitrary Waveform Generator (AWG), using an over-coupled ER

5106 QT2 resonator (Bruker) at Q-band (34 GHz). The temperature was maintained at 60 K using liquid helium and a CF935 cryostat (Oxford Instruments) with an ITC103 temperature controller (Oxford Instruments). Laser excitation was provided by the second harmonic (532 nm) of a Nd:YAG laser (Continuum, Surelite I) operated at a repetition rate of 10 Hz (7 ns pulses), with an energy of ca. 2 mJ per pulse. The beam was passed through a depolariser before reaching the window of the cryostat.

ReLaserIMD experiments at Q-band used a refocused-echo pulse sequence ($\pi/2 - \tau_1 - \pi - \tau' -$ laser $- \tau'' - \pi - \tau_2 -$ echo) with rectangular $\pi/2$ (20 ns) and π (40 ns) pulses and time delays $\tau_1 =$ 800 ns and $\tau_2 = 200$ ns, $\tau_1 + \tau_2 = \tau' + \tau''$. Traces were acquired using a time step of 4 ns.

Electron spin-echo (ESE) experiments (i.e. field sweeps, phase-memory-time and inversion-recovery experiments) were performed using a standard Hahn echo sequence ($\pi/2 - \tau - \pi - \tau$ – echo), with lengths of 40 and 20 ns for the $\pi/2$ and π pulses, respectively, and a τ value of 200 ns. The spin Hamiltonian parameters used to simulate the EPR spectrum of the nitroxide radical were obtained from fitting the echo-detected field-swept spectrum (Fig. 2 (a)), using the Matlab® *EasySpin* routine (*pepper* function).[3]

trEPR experiments at Q-band (34 GHz) were conducted with depolarised light were conducted in an Elexsys E580 spectrometer (Bruker) using a critically-coupled ER 5106 QT2 resonator (Bruker). Laser excitation was provided by the second harmonic (532 nm) of a Nd:YAG laser (Continuum, Surelite I) operated at a repetition rate of 10 Hz (7 ns pulses), with an energy of ca. 2 mJ per pulse. The beam was passed through a depolariser before reaching the window of the cryostat.

Experiments with polarised light

Pulsed experiments with polarised light were conducted at X-band (9.5 GHz) in an Elexsys E580 spectrometer (Bruker) equipped with a Bruker dielectric resonator (ER4118X-MD5(W1)) and a CF935 cryostat (Oxford Instruments). Measurements were performed at 80 K using a nitrogen vapour flow and an ITC503 temperature controller (Oxford Instruments). The sample was photoexcited with a pulsed Nd:YAG laser (Quantel Brilliant) equipped with the second harmonic module, operated at a repetition rate of 10 Hz (5 ns pulses) with an energy of ca. 3.5 mJ/pulse.

trEPR experiments at X-band (9.5 GHz) were carried out in an Elexsys E580 spectrometer (Bruker) equipped with a critically coupled dielectric resonator (ER4118X-MD5(W1)), measuring in direct detection mode, without field modulation or phase sensitive detection. Measurements were performed at 80 K using a nitrogen gas flow, an Oxford CF900 cryostat and an Oxford ITC504 temperature controller. The signal was collected from the microwave amplifier (bandwidth 20Hz – 6.5 MHz) and sampled with a LeCroy 9360 oscilloscope. The microwave power for the experiments was set at 0.6 mW. The sample was photoexcited with a pulsed Nd:YAG laser (Quantel Rainbow) equipped with an optical parametric oscillator (OPO) pumped by the third harmonic module (excitation wavelength = 525 nm), operating at a repetition rate of 10 Hz, with light linearly polarised parallel and perpendicular to the static magnetic field. Laser pulses were 5 ns long with energy of 1 mJ per pulse.

The rotation of the polarisation of the light for magnetophotoselection experiments was obtained with a half-wave plate and a Glan-Thompson prism for better control. Spectra free from magnetophotoselection were obtained by adding together the spectrum obtained with

light polarised parallel to the static magnetic field and the spectrum obtained with light polarised perpendicular to the static magnetic field multiplied by two (Equation 3). Triplet state parameters for I_2BODIPY were extracted via simulation of trEPR spectra using a home-written MATLAB® program. [4]

ReLaserIMD experiments at X-band used a refocused echo pulse sequence with 40 ns pulses, time delays τ_1 = 620 ns and τ_2 = 260 ns. Traces were acquired using a time step of 4 ns.

It was observed that the I_2BODIPY chromophore was stable in the EPR experiments for a period of several days without significant degradation under the illumination conditions.

S2. COMPUTATIONAL METHODS

S2.1. DFT calculations

Initial geometries for molecule **1** were built based on previous DFT calculations,[2] using UCSF Chimera.[5] Geometry optimizations and spin density calculations were performed *in vacuo* using Gaussian® 16 (revision A.03).[6] Ground state geometry optimizations were carried out using the PBE1PBE functional and the 6-31g(d) basis set. Iodine atoms were replaced by hydrogens to speed up the calculation.

The geometry optimization and spin density calculation of I_2BODIPY-carboxy in the triplet state (spin multiplicity of 3) was carried out in Orca (release version 4.2.0),[7] using the

functional B3LYP and the basis set Def2-TZVP. The RIJCOSX approximation, with the auxiliary basis set def2/J, was used. The ZFS tensor orientation of the I_2BODIPY-carboxy triplet was calculated using the basis set EPR-II[8] for H, C, N, O, B and F, and Def2-TZVP for I. The spin-spin contribution to the ZFS was calculated using computed spin-unrestricted natural orbital (UNO) determinants, and no spin-orbit contribution was included.[9]

S2.2. Orientation-dependent ReLaserIMD simulations with depolarised light

The orientation-dependent simulation protocol first described for conventional PDS by Lovett *et al.*[10] and later adapted to LiPDS by Bowen *et al.*[11] was used for the ReLaserIMD simulations with depolarized light. Simulations were carried out in the g-tensor frame of the nitroxide radical (Fig. 1). The pump pulse was considered to excite all orientations of the I_2BODIPY chromophore with respect to the external magnetic field, reflecting the fact that the laser light used in this experiment was depolarized. The spin Hamiltonian parameters used to simulate the EPR spectra of the nitroxide radical and I_2BODIPY triplet were obtained from fitting the echo-detected field-swept (Fig. S6) and trEPR (Fig. S7) spectra, respectively, using the Matlab® *EasySpin* routine (*pepper* function).[3]

A library of pre-simulated traces was fitted to the experimental data following the protocol reported by Marko et al.[12] For the model-based fit, an initial model consisting of a cone of 1000 dipolar vectors around the DFT-optimised geometry was generated ($\Delta\theta = \Delta\phi = 30°,\ \Delta r = \ 0.2\ nm$), where the orientation of the chromophore was varied according to the Euler angles α, β and γ that define the orientation of the ZFS-tensor frame of the I_2BODIPY triplet with respect to the g-tensor

frame of the nitroxide radical ($\Delta\alpha = \Delta\beta = \Delta\gamma = 20°$). The corresponding dipolar traces at the 9 different field positions were simulated taking into account the spin density delocalization in the I_2BODIPY triplet as calculated by DFT (Fig. S9). A second library of dipolar traces was calculated from a finer set of 1000 dipolar vectors around the best fitting structure of the first model ($\Delta\theta = \Delta\phi = 15°,\ \Delta r = 0.2\ nm,\ \Delta\alpha = \Delta\beta = \Delta\gamma = 10°$), and it was used to fit the experimental data again (Fig. 2 (b)). For the model-free fit, a set of ca. 10000 dipolar vectors over one quarter of a spherical shell using a spherical grid with knots evert 10° with $r = 1.5\ nm$ to $2.1\ nm$ were employed to simulate the library of dipolar traces used for the fit to the experimental data, and all the electron spin density of the I_2BODIPY triplet was considered to be concentrated at the centre of the chromophore, whose orientation was kept constant. A total of 50 least-squares fitting iterations was sufficient to reach convergence of the root-mean-square deviation (RMSD) from the experimental data (Fig. S11).

S2.3. Orientation-dependent ReLaserIMD simulations with linearly polarised light

Simulations used a modified version of the previously published algorithm for simulating orientation dependent traces.[11] This algorithm relies on calculating the overlap between bandwidth of the pulses used for detection and pump and the EPR spectrum of the spin active center upon which the pules acts. For LaserIMD, the detection pulses act on a permanent spin-center in the same way as would be expected for DEER. In unpolarized LaserIMD the laser pump pulse is modelled to have infinite uniform bandwidth exciting all spin centers in a non-polarized triplet spectrum equally no matter the orientation with respect to the magnetic field.

In the case of the use of polarized light this is incorporated into the calculation by using a simulated polarized triplet spectrum. The polarization is modelled using the exp.ordering parameter written into EasySpin, using the functions and parameters defined in the main paper.[13]

Simulations used the five best fitting conformations from the model free fitting procedure described in Section S2.2, which were seen to cover 58% of the conformational space occupied by the fitted geometries. This provided five sets of spherical polar coordinates (θ, ϕ, r), which each have a relative weight out of 50 fitted conformations, see Table S1. For each of these sets of polar coordinates traces were calculated with Euler angles (α, β, γ) generated by a grid of knots with C1 symmetry and 5 knots per quarter meridian to generate a library of traces.

Table S1. Spherical polar coordinates describing five best fitting orientations from the model based fit to the Q-band data which were used as a basis for the orientation dependent magnetophotoselective simulations.

**Identifier**	**Relative weight**	**φ**	**θ**	**r**
1	7	-2.0494	0.3333	1.6956
2	6	-2.3473	0.4180	1.7469
3	6	-2.2944	0.1975	1.7114
4	5	-2.4881	0.3817	1.7584
5	5	-1.8908	0.2063	1.9025

Simulated traces were filtered according to the ordering of the calculated relative modulation depths and only those that agreed with the observed experimental order were considered acceptable as described in Section S3.4. Euler angle orientations corresponding to these are plotted in Fig. S15.

S3. RESULTS

S3.1. Characterization

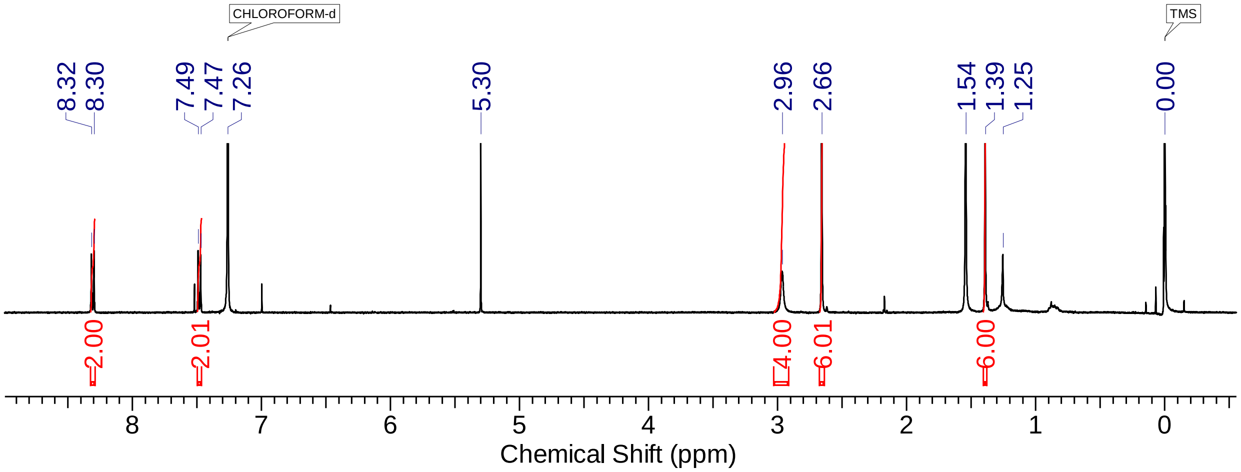

Figure S1. ^{1}H NMR spectrum of compound I_2BODIPY-O*N*Su ($CDCl_3$, 400 MHz).

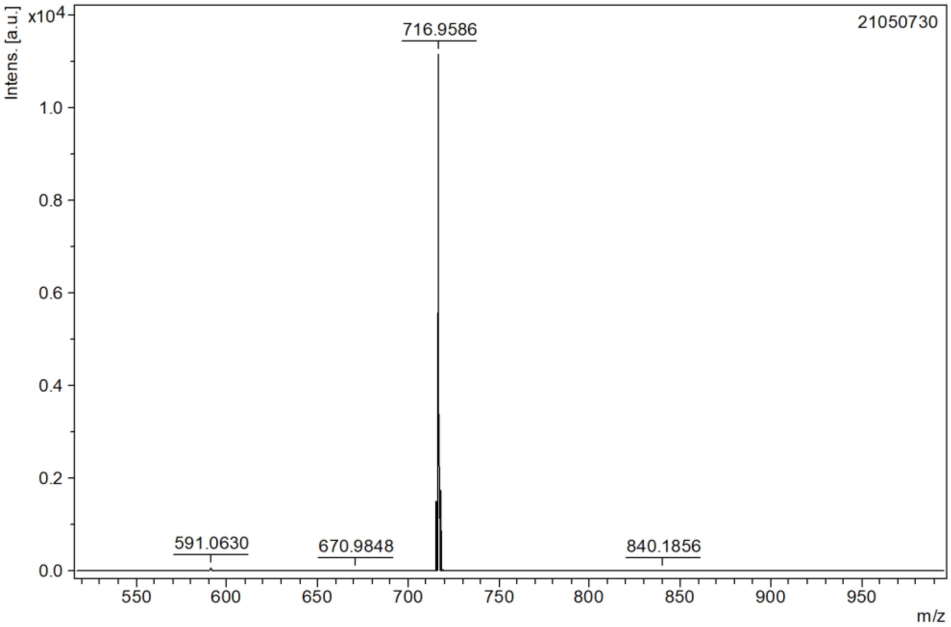

Figure S2. MALDI–TOF high resolution mass spectrum of compound I_2BODIPY-O*N*Su.

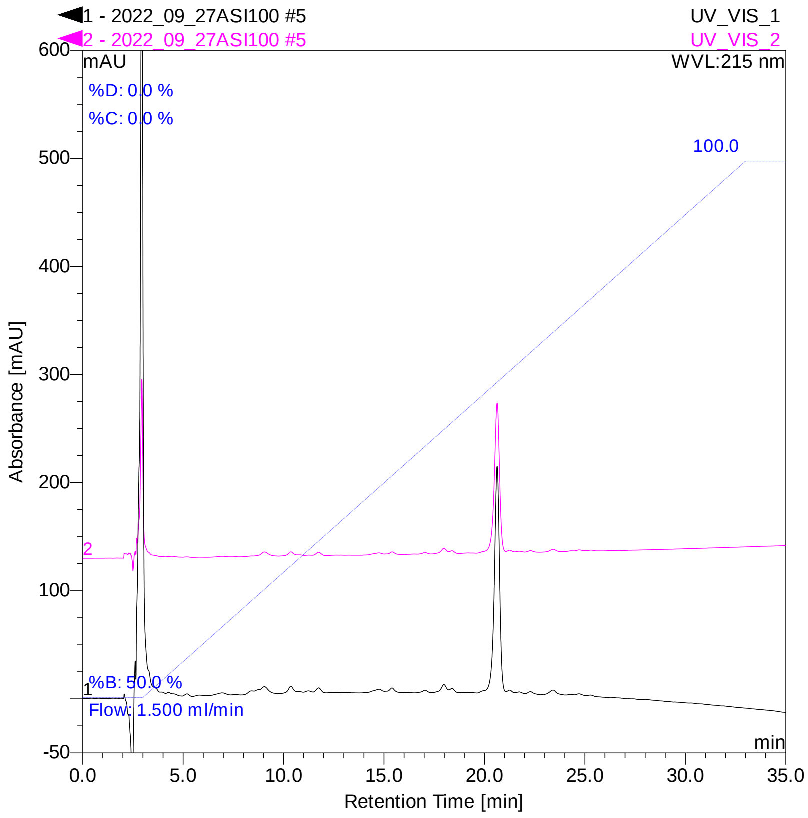

Figure S3. HPLC chromatogram of **1**.

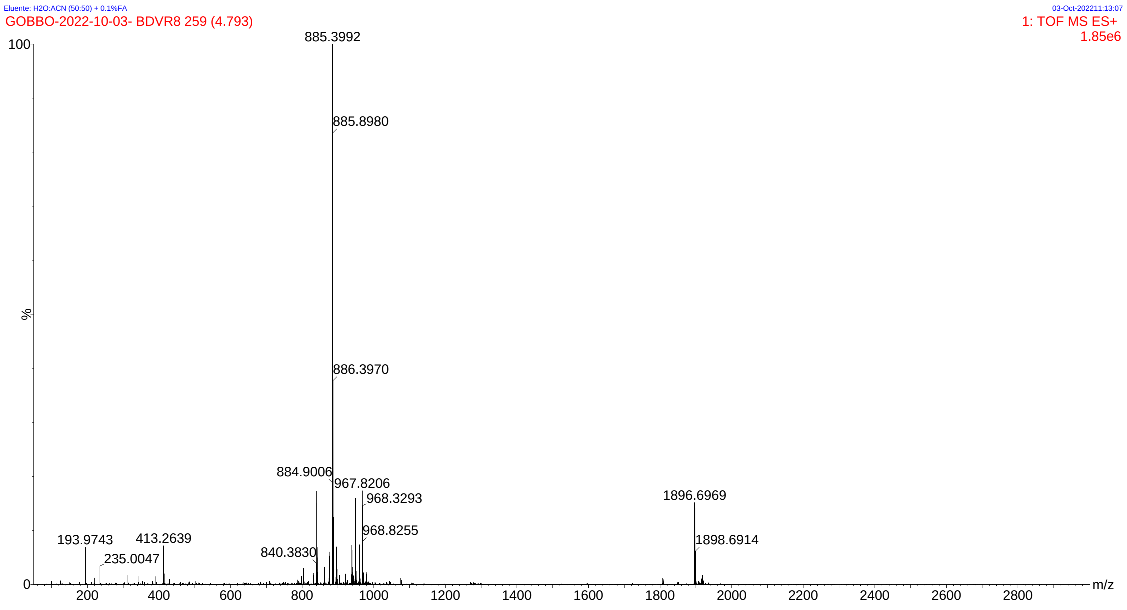

Figure S4. ESI-MS spectrum of **1**.

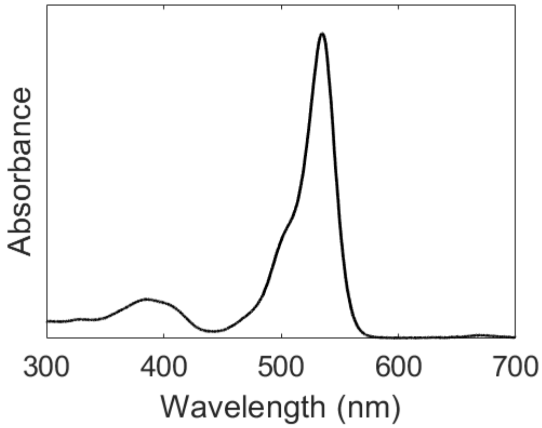

Figure S5. Room-temperature UV-Vis absorption spectrum of **1** in d_4-methanol:d_6-ethanol 3:2 (v:v). Spectrum measured using a Cary60 UV-Vis spectrometer (Agilent) and a quartz cuvette.

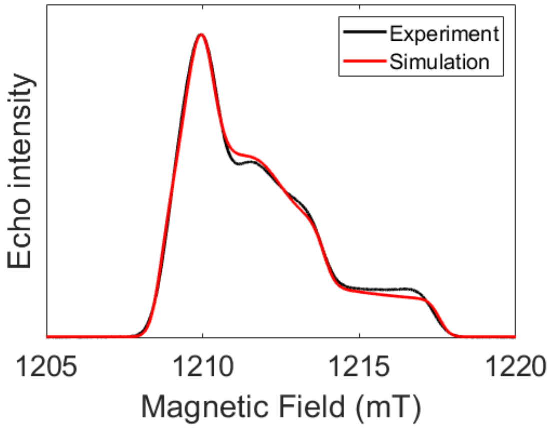

Figure S6. Electron spin-echo field-swept spectrum of **1** in the dark (black) and simulation (red) using the Matlab® *EasySpin* routine (*pepper* function)[3]: g = [2.0086, 2.0052, 2.0014], g-strain = [0.0011, 0.0020, 0.0011], A_N = [2.17, 13.8, 103.2] MHz and A-strain = [0, 0, 1] mT.

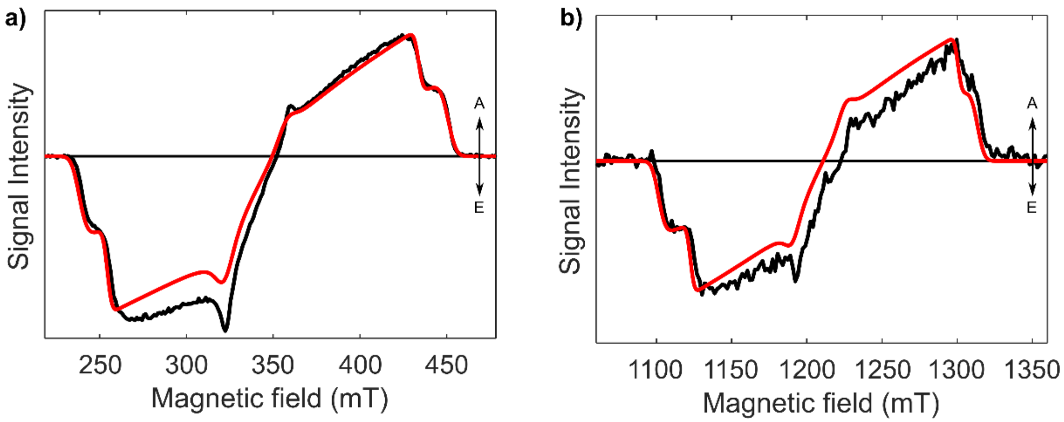

Figure S7. Isotropic trEPR spectra of **1** after photoexcitation at 532 nm at X-band (a) and Q-band (b) (black), obtained by averaging together the spectrum obtained with light polarized parallel to the static magnetic field and the spectrum obtained with light polarized perpendicular to the static magnetic field multiplied by two. Simulation (red) using the Matlab® *EasySpin* routine (*pepper* function)[3]. Simulation parameters: g = [2.0036, 2.0079, 2.0112], D = -2986 MHz, E = 667 MHz, H-strain = [122, 229, 229] MHz, linewidth = [2.08, 0.03] mT and triplet state sublevel populations p_x = 0.00, p_y = 0.18 and p_z = 0.82. These values are in agreement with previous studies [14].

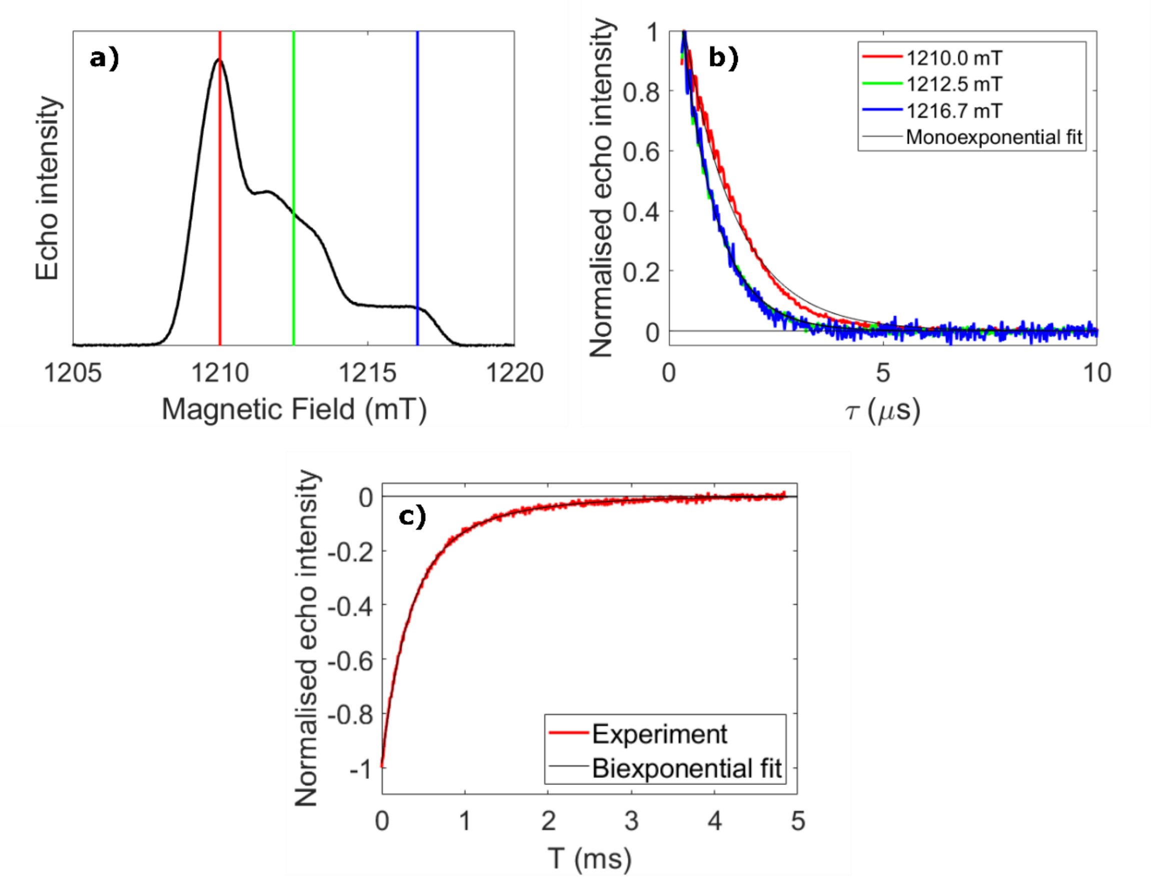

Figure S8. Q-band pulsed EPR characterization of **1** in the dark. (a) Electron spin echo field swept spectrum, with the field positions used for phase-memory time experiments indicated as vertical lines. (b) Electron spin phase-memory time experiments at the field positions indicated in (a) (coloured lines) and monoexponential fits (black lines) rendering T_m values of (1.22 ± 0.02) μs, (0.818 ± 0.009) μs and (0.80 ± 0.01) μs, from left to right. (c) Inversion-recovery experiment performed at the signal maximum (red) and biexponential fit (black) with lifetimes of (0.315 ± 0.008) μs and (1.03 ± 0.04) μs.

S3.2. DFT results

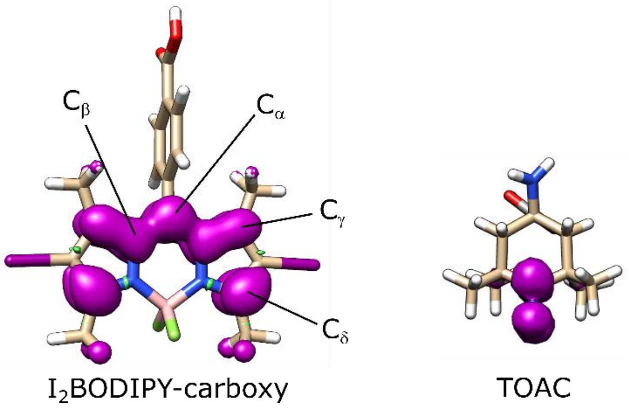

Figure S9. Calculated electronic spin densities for the two labels (I_2BODIPY-carboxy triplet, left; TOAC nitroxide radical, right) used in the orientation-dependent analysis. See section S2.1. for the computational details.

Table S2. I_2BODIPY triplet electronic spin density values (taken as Mulliken atomic spin densities) used for the orientation-dependent ReLaserIMD simulations. Labelling according to Fig. S9.

Atom	Spin density
C_α	0.3200
C_β	0.2230
C_γ	0.2150
C_δ	0.4010

Table S3. Nitroxide radical (TOAC) electronic spin density values (taken as Mulliken atomic spin densities) used for the orientation-dependent ReLaserIMD simulations.

Atom	Spin density
N	0.4437
O	0.5200

S3.3. Orientation-selective ReLaserIMD with depolarised light

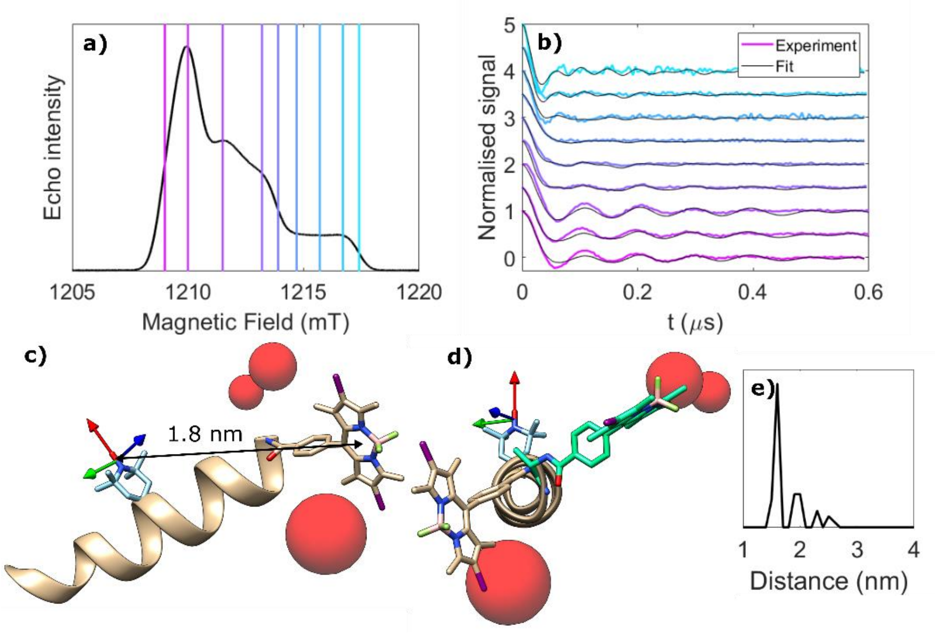

Figure S10. Model-free analysis of the orientationally selective ReLaserIMD on **1**. (a) Electron spin-echo field-swept spectrum in the dark, showing the magnetic field values where ReLaserIMD traces were acquired. (b) Background-corrected and modulation depth-normalised ReLaserIMD traces (coloured lines) and orientation-dependent model-free fits (black lines). (c) DFT-optimised structure of **1** showing the different positions of the I_2BODIPY centre determined by the fitting procedure as red spheres, relative to the nitroxide g-tensor frame (arrows: red = g_x, green = g_y, blue = g_z). The diameter of the spheres is proportional to the number of times a single I_2BODIPY position contributes to the fit shown in panel (b). (d) Projection view of panel (c), including a second local energy minimum conformation (+ 9 kJ/mol) identified by DFT (green). (e) Spin–spin distance distribution obtained from the orientation-dependent analysis.

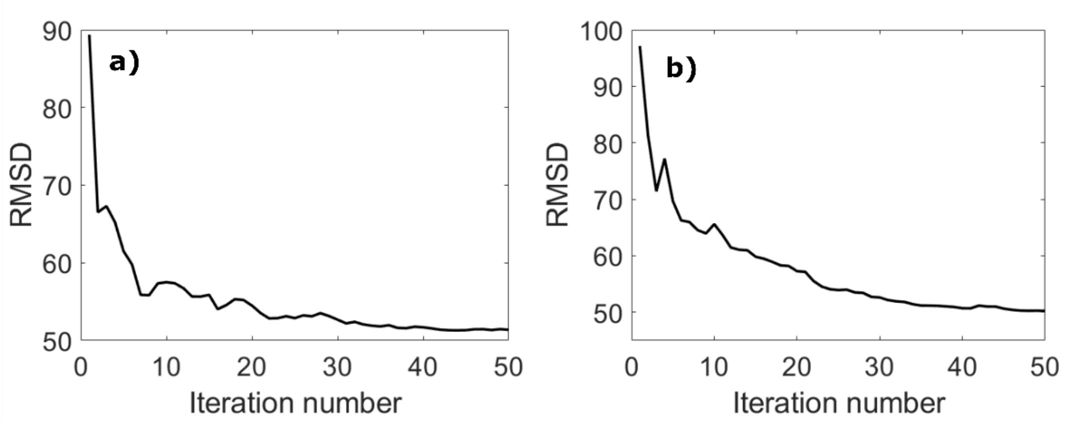

Figure S11. Root-mean-square deviation plots for the model-based (a) and model-free (b) orientational fits to the ReLaserIMD dataset measured with depolarised light.

Table S4. Details for the ReLaserIMD traces measured with depolarised light.

Magnetic field [mT]	Scans	Modulation depth	Noise level (stdev)	Modulation-to-noise ratio (MNR)
1209.0	800	0.04	8.5 $\times10^{-4}$	47
1210.0	200	0.03	9.1$\times10^{-4}$	33
1211.5	400	0.05	10.0 $\times10^{-4}$	48
1213.2	800	0.02	5.2 $\times10^{-4}$	77
1213.9	1400	0.04	7.4 $\times10^{-4}$	54
1214.7	2400	0.05	9.4 $\times10^{-4}$	53
1215.7	5200	0.01	9.5 $\times10^{-4}$	11
1216.7	2565	0.03	11.0 $\times10^{-4}$	27
1217.4	2600	0.02	16.0 $\times10^{-4}$	13

S3.4. Magnetophotoselection ReLaserIMD

Table S5. Simulation parameters of TR-EPR spectra obtained both with polarised light and with isotropic excitation: ZFS parameters D and E; relative triplet sublevel populations p_i (i = X,Y,Z); polar angles ω (angle between the TDM and the Z ZFS axis) and φ (angle between the X ZFS axis and the projection of the direction of the TDM into the XY ZFS plane) describing the orientation of the TDM with respect to the ZFS axes tensor; isotropic percentage contribution to the MPS trEPR simulation c_{iso}.

|D| [MHz]	|E| [MHz]	$p_X:p_Y:p_Z$	ω (°)	φ (°)	c_{iso} (%)
2985	667	0:0.17:0.83	22	0	37

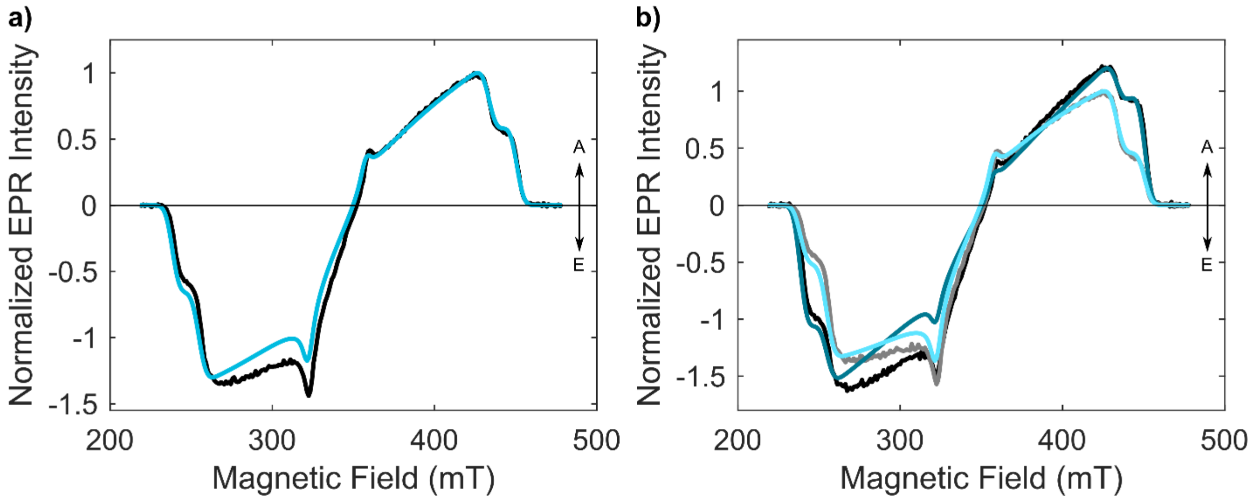

Figure S12. Simulation of the trEPR spectra of **1** with TESEO. (a) Magnetophotoselection-free trEPR spectrum (black), normalised to its maximum, with the corresponding simulation (blue), obtained by adding twice the spectrum obtained with light polarised perpendicular to the static magnetic field to the one obtained with light polarised parallel to the static magnetic field. (b) Experimental trEPR spectra obtained with light polarised parallel (black) and perpendicular (grey) to the magnetic field, both normalised to the maximum of the one recorded with perpendicular polarisation of the light, and the corresponding simulations (darker and lighter blue, respectively). Spectra extracted at the maximum of the triplet signal. Simulation parameters are reported in Table S5.

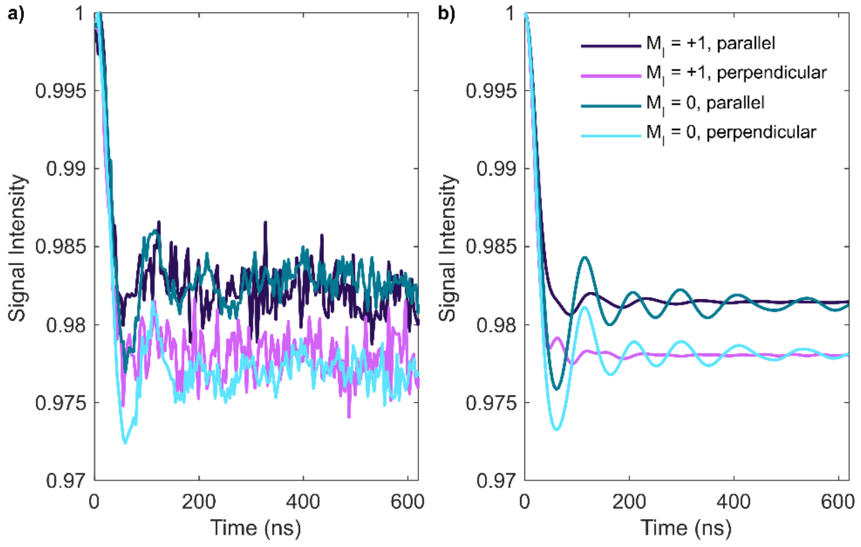

Figure S13. ReLaserIMD with polarised light. (a) Experimental ReLaserIMD form factors obtained with polarised light, with modulation depths corrected by the factors obtained from the magnetophotoselection-free form factors. (b) Corresponding simulated form factors, following the condition for their relative modulation depths (Δ): $\Delta(m_I=0,\perp)>\Delta(m_I=+1,\perp)>\Delta(m_I=+1,\parallel)>\Delta(m_I=0,\parallel)$.

Table S6. Details for the ReLaserIMD traces measured with polarised light.

Magnetic field [mT]	Light polarisation*	Microwave Frequency [GHz]	Scans	Modulation depth	Noise level (stdev)	Modulation-to-noise ratio (MNR)
342.8	Parallel	9.720	2820	0.02	14 x10^{-4}	14
	Perpendicular			0.02	16 x10^{-4}	13
345.3	Parallel	9.715	1860	0.02	8.9 x10^{-4}	22
	Perpendicular			0.02	10 x10^{-4}	20

*Direction with respect to the magnetic field

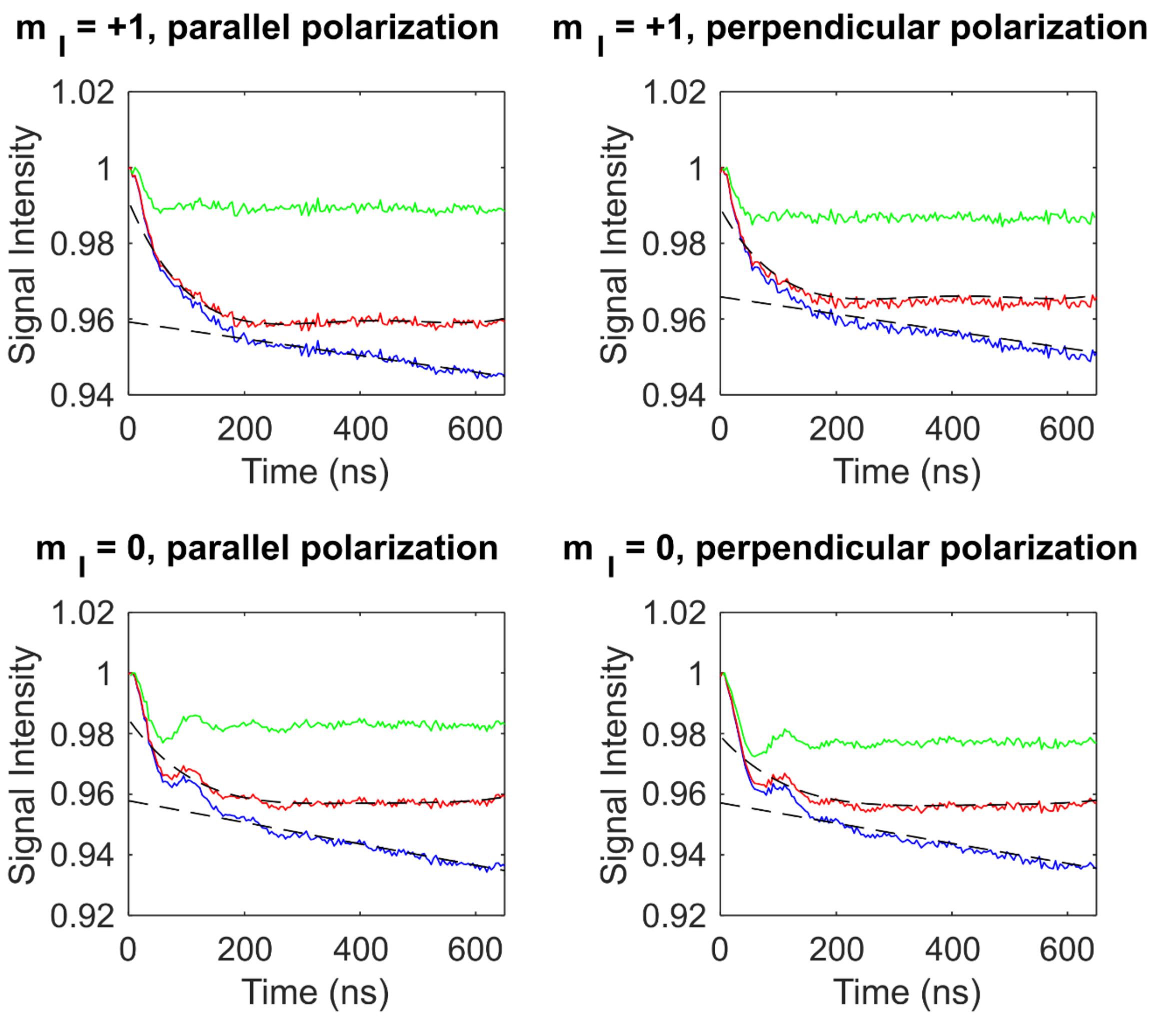

Figure S14. Background subtraction for ReLaserIMD data of **1** with polarised light in X-Band. Blue: experimental trace; red: experimental trace after subtraction of a 3-dimensional exponential background; green: experimental trace after successive subtraction of a 4th-order polynomial background function; black, dashed: background functions.

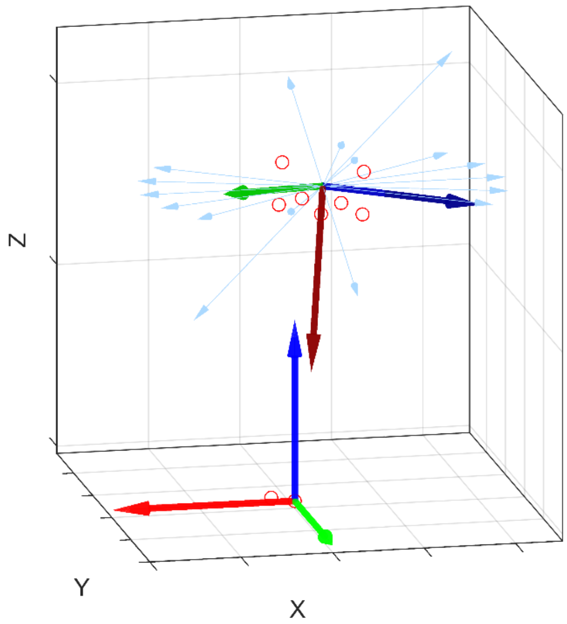

Figure S15. Allowed orientations for the Z- ZFS axes for I_2BODIPY (light blue) with respect to the g-tensor of the nitroxide (bottom arrows: red = g_x, green = g_y, blue = g_z; upper arrows: initial orientation of the ZFS tensor in the optimised structure, dark red = X, dark green = Y, dark blue = Z). Red circles: initial position of nuclei where the I_2BODIPY spin density is mostly localised.

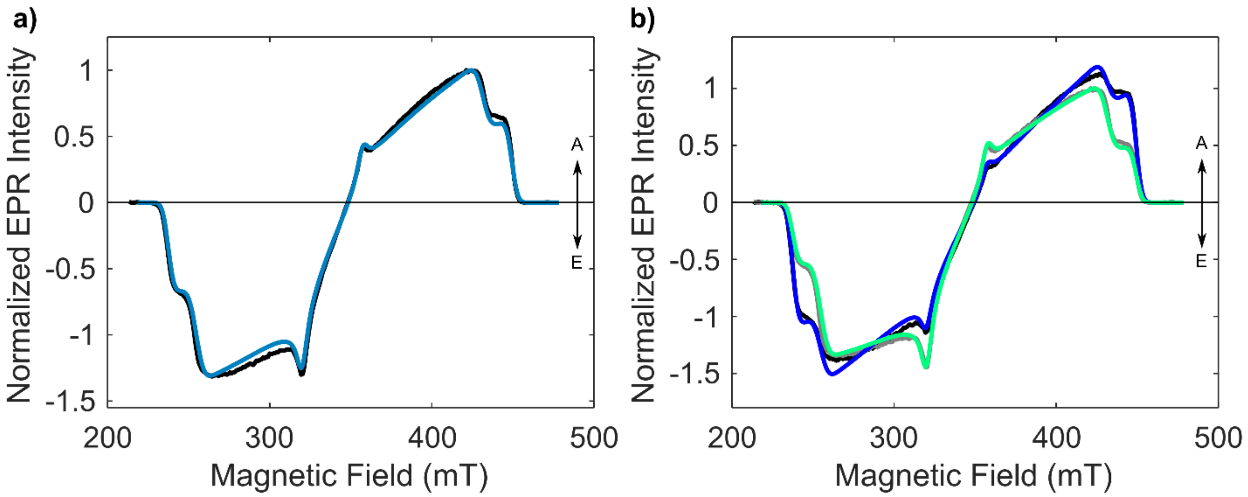

Figure S16. Simulation of the trEPR spectra of I_2BODIPY with TESEO. (a) Magnetophotoselection-free trEPR spectrum (black), normalised to its maximum, with the corresponding simulation (blue), obtained by adding twice the spectrum obtained with light polarised perpendicular to the static magnetic field to the one obtained with light polarised parallel to the static magnetic field. (b) Experimental trEPR spectra obtained with light polarised parallel (black) and perpendicular (grey) to the magnetic field, both normalised to the maximum of the one recorded with perpendicular polarisation of the light, and the corresponding simulations (blue and green, respectively). Spectra extracted at the maximum of the triplet signal. Simulation parameters: g = [1.99905 2.00484 2.00779], D = -2974 MHz, E = 647 MHz, gaussian linewidth = [36, 30, 28] mT, triplet state sublevel populations p_x = 0.00, p_y = 0.16 and p_z = 0.84, ω (°) = 22°, φ (°) = 0°, c_{iso} (%) = 40.

REFERENCES

1. Guo, S.; Zhang, H.; Huang, L.; Guo, Z.; Xiong, G.; Zhao, J. Porous Material-Immobilized Iodo-Bodipy as an Efficient Photocatalyst for Photoredox Catalytic Organic Reaction to Prepare Pyrrolo[2,1-a]Isoquinoline. *Chem. Commun.* **2013**, *49*, 8689–8691, doi:10.1039/c3cc44486d.

2. Di Valentin, M.; Albertini, M.; Dal Farra, M.G.; Zurlo, E.; Orian, L.; Polimeno, A.; Gobbo, M.; Carbonera, D. Light-Induced Porphyrin-Based Spectroscopic Ruler for Nanometer Distance Measurements. *Chem. - A Eur. J.* **2016**, *22*, 17204–17214, doi:10.1002/chem.201603666.

3. Stoll, S.; Schweiger, A. EasySpin, a Comprehensive Software Package for Spectral Simulation and Analysis in EPR. *J. Magn. Reson.* **2006**, *178*, 42–55, doi:10.1016/j.jmr.2005.08.013.

4. Toffoletti, A.; Wang, Z.; Zhao, J.; Tommasini, M.; Barbon, A. Precise Determination of the Orientation of the Transition Dipole Moment in a Bodipy Derivative by Analysis of the Magnetophotoselection Effect. *Phys. Chem. Chem. Phys.* **2018**, *20*, 20497–20503, doi:10.1039/C8CP01984C.

5. Pettersen, E.F.; Goddard, T.D.; Huang, C.C.; Couch, G.S.; Greenblatt, D.M.; Meng, E.C.; Ferrin, T.E. UCSF Chimera - A Visualization System for Exploratory Research and Analysis. *J. Comput. Chem.* **2004**, *25*, 1605–1612, doi:10.1002/jcc.20084.

6. Frisch, M.J.; Trucks, G.W.; Schlegel, H.B.; Scuseria, G.E.; Robb, M.A.; Cheeseman, J.R.; Scalmani, G.; Barone, V.; Petersson, G.A.; Nakatsuji, H.; et al. Gaussian 09 2009.

7. Neese, F. The ORCA Program System. *WIREs Comput. Mol. Sci.* **2012**, *2*, 73–78, doi:10.1002/wcms.81.

8. Barone., V. Structure, Magnetic Properties and Reactivities of Open-Shell Species from Density Functional and Self-Consistent Hybrid Methods. In *Recent Advances in Density Functional Methods, Part I*; Chong, D.P., Ed.; World Scientific Publ. Co.: Singapore, 1996.

9. Sinnecker, S.; Neese, F. Spin-Spin Contributions to the Zero-Field Splitting Tensor in Organic Triplets, Carbenes and Biradicals - A Density Functional and Ab Initio Study. *J. Phys. Chem. A* **2006**, *110*, 12267–12275, doi:10.1021/jp0643303.

10. Lovett, J.E.; Bowen, A.M.; Timmel, C.R.; Jones, M.W.; Dilworth, J.R.; Caprotti, D.; Bell, S.G.; Wong, L.L.; Harmer, J. Structural Information from Orientationally Selective DEER Spectroscopy. *Phys. Chem. Chem. Phys.* **2009**, *11*, 6840–6848, doi:10.1039/b913085n.

11. Bowen, A.M.; Bertran, A.; Henbest, K.B.; Gobbo, M.; Timmel, C.R.; Di Valentin, M. Orientation-Selective and Frequency-Correlated Light-Induced Pulsed Dipolar Spectroscopy. *J. Phys. Chem. Lett.* **2021**, *12*, 3819–3826, doi:10.1021/acs.jpclett.1c00595.

12. Marko, A.; Prisner, T.F. An Algorithm to Analyze PELDOR Data of Rigid Spin Label Pairs. *Phys. Chem. Chem. Phys.* **2012**, *15*, 619–627, doi:10.1039/C2CP42942J.

13. Tait, C.E.; Krzyaniak, M.D.; Stoll, S. Computational Tools for the Simulation and Analysis of Spin-Polarized EPR Spectra. *J. Magn. Reson.* **2023**, *349*, 107410, doi:10.1016/j.jmr.2023.107410.

14. Wang, Z.; Sukhanov, A.A.; Toffoletti, A.; Sadiq, F.; Zhao, J.; Barbon, A.; Voronkova, V.K.; Dick, B. Insights into the Efficient Intersystem Crossing of Bodipy-Anthracene Compact Dyads with Steady-State and Time-Resolved Optical/Magnetic Spectroscopies and Observation of the Delayed Fluorescence. *J. Phys. Chem. C* **2019**, *123*, 265–274, doi:10.1021/acs.jpcc.8b10835.
